# Supplementary material for: A Likelihood Approach for Real-Time Calibration of Stochastic Compartmental Epidemic Models
Source: PLoS Comput Biol. 2017 Jan 17;13(1):e1005257. doi: 10.1371/journal.pcbi.1005257 (PMC5240920; doi:10.1371/journal.pcbi.1005257)
Supplement: S1 File — (TAR.GZ) [file pcbi.1005257.s014.tar.gz › HSPH_Online-SI-Revision/Additions/MSS11/n100-extreme_noisy/n100-extreme_noisy_NEW_table4.pdf]

1	R0	{2.76052, 16.3913, 0.1}	{2.98125, 18.4749, 2.}	{{2.98125, 18.4749, 2.}, {2.98125, 18.4749, 2.}}	{7.99606, 12.7113, 1900.}	inf	11577.	13297.3	{13198.2, 13396.7}	14.8598	0.0601616	newly	4368.49	4421.95	{4337., 4499.}	1.50178	0.00645861	Reff	0.335762	0.305358	{0.30367, 0.306809}	9.05531	ar	92090.	93907.8	{93807., 94010.}	1.97397
2	R0	{2.18202, 15.1734, 3.}	{2.22867, 15.6411, 3.}	{{2.22867, 15.6411, 3.}, {2.22867, 15.6411, 3.}}	{2.13793, 3.08213, 0.}	inf	9523.	9700.14	{9607.95, 9808.47}	1.86016	0.00799212	newly	3796.	3866.18	{3771., 3949.}	2.14067	0.0091729	Reff	0.491915	0.482354	{0.480951, 0.483836}	1.94356	ar	84264.	84949.9	{84787., 85115.}	0.814025
3	R0	{2.57891, 12.5971, 1.}	{2.53075, 11.6597, 0.}	{{2.53075, 11.6597, 0.}, {2.53075, 11.6597, 0.}}	{1.86748, 7.44128, 100.}	inf	6810.	5767.67	{5667.04, 5846.67}	15.3058	0.0721729	newly	3181.	2834.17	{2773., 2895.}	10.9032	0.0501976	Reff	0.320791	0.32157	{0.320412, 0.322746}	0.307467	ar	90302.	89648.5	{89535., 89734.}	0.723694
4	R0	{2.5856, 9.28508, 0.1}	{2.93493, 10.3791, 0.01}	{{2.93906, 10.3908, 0.}, {2.93906, 10.3908, 0.}}	{13.5562, 11.7964, 108.}	inf	4198.	4868.31	{4807.36, 4948.63}	16.1038	0.0648237	newly	2428.	2549.79	{2500., 2610.}	5.16021	0.0218042	Reff	0.287622	0.225668	{0.223695, 0.226043}	21.6321	ar	90312.	93564.8	{93523., 93677.}	3.61875
5	R0	{2.4406, 12.1341, 1.}	{2.58092, 13.7736, 2.}	{{2.58092, 13.7736, 2.}, {2.58092, 13.7736, 2.}}	{5.7495, 13.5117, 100.}	inf	8911.	10593.	{10492.2, 10686.}	18.8752	0.0750804	newly	4440.	4593.23	{4504., 4670.}	3.46869	0.0147685	Reff	0.386079	0.372987	{0.371602, 0.374328}	3.39094	ar	88597.	90255.5	{90135., 90378.}	1.87193
6	R0	{2.72155, 15.4094, 2.}	{2.93371, 17.1903, 1.}	{{2.93371, 17.1903, 1.}, {2.93371, 17.1903, 1.}}	{7.79541, 11.5572, 50.}	inf	11194.	12819.8	{12714.4, 12922.4}	14.5235	0.0588852	newly	4357.	4536.01	{4470., 4619.}	4.11177	0.0174646	Reff	0.34629	0.31012	{0.308106, 0.311602}	10.4451	ar	91682.	93570.4	{93470., 93665.}	2.05971
7	R0	{2.63781, 14.3935, 0.1}	{2.92797, 18.0614, 0.}	{{2.96797, 18.4042, 0.}, {2.96797, 18.4042, 0.}}	{12.0304, 25.788, 100.}	inf	9306.	13020.9	{13182.8, 13452.5}	40.2506	0.145222	newly	3953.	4405.4	{4331., 4518.}	11.8811	0.0486202	Reff	0.327906	0.314999	{0.307451, 0.311083}	6.69517	ar	91192.	93420.2	{93694., 93886.}	2.85245
8	R0	{2.82391, 16.104, 1.}	{2.93371, 17.1903, 1.}	{{2.93371, 17.1903, 1.}, {2.93371, 17.1903, 1.}}	{3.88808, 6.74551, 0.}	inf	11648.	12825.5	{12700.8, 12937.7}	10.1087	0.0418123	newly	4404.	4540.39	{4452., 4617.}	3.10241	0.0132278	Reff	0.322067	0.309869	{0.308174, 0.311591}	3.78731	ar	92664.	93574.8	{93477., 93661.}	0.982928
9	R0	{2.80645, 14.6457, 0.1}	{2.7499, 15.3124, 0.}	{{2.7499, 15.3124, 0.}, {2.7499, 15.3124, 0.}}	{2.0151, 4.55228, 100.}	inf	11375.	13081.8	{12990.3, 13171.2}	15.0045	0.0607082	newly	4649.	5165.98	{5088., 5251.}	11.1202	0.0457632	Reff	0.325885	0.36214	{0.360331, 0.364171}	11.1252	ar	92567.	92035.6	{91914., 92136.}	0.574027
10	R0	{1.86213, 5.4271, 0.1}	{1.81768, 5.68021, 1.}	{{1.81768, 5.68021, 1.}, {1.81768, 5.68021, 1.}}	{2.38702, 4.66374, 900.}	inf	1169.	1346.53	{1290.99, 1404.42}	15.1863	0.0611584	newly	1101.	1235.29	{1175., 1292.}	12.1971	0.0496958	Reff	0.480541	0.497355	{0.495877, 0.498472}	3.49887	ar	75362.	73905.3	{73739., 74076.}	1.93298
11	R0	{2.27349, 14.6338, 1.}	{2.20937, 14.0906, 2.97}	{{2.20345, 14.058, 3.}, {2.20345, 14.058, 3.}}	{3.15634, 3.92195, 197.}	inf	9305.	9002.3	{8874.77, 9100.16}	3.49699	0.0154523	newly	3974.	3921.56	{3834., 3997.}	1.63664	0.00719161	Reff	0.44615	0.476578	{0.476608, 0.479894}	7.18939	ar	85805.	84559.	{84290., 84637.}	1.58634
12	R0	{2.77261, 14.1894, 0.1}	{2.73108, 15.6124, 0.}	{{2.73108, 15.6124, 0.}, {2.73108, 15.6124, 0.}}	{1.4977, 10.0288, 100.}	inf	10089.	11388.2	{11314.4, 11467.8}	12.8773	0.0525996	newly	4154.	4409.39	{4348., 4481.}	6.14805	0.0258771	Reff	0.306207	0.341704	{0.340228, 0.343031}	11.5926	ar	92508.	91856.9	{91746., 91965.}	0.703885
13	R0	{2.65402, 19.3935, 2.}	{2.23826, 14.6301, 1.77}	{{2.08003, 13.1443, 1.}, {2.76795, 19.6041, 2.}}	{17.6401, 25.0616, 11.5}	inf	15461.	11012.1	{9166.81, 16884.3}	32.9597	0.180021	newly	4998.	4605.14	{4304., 5430.}	11.7551	0.0537033	Reff	0.43088	0.51472	{0.423062, 0.543701}	20.2909	ar	91184.	84123.7	{81531., 92198.}	8.25749
14	R0	{1.75297, 4.45354, 0.1}	{2.22376, 9.35432, 0.}	{{2.22376, 9.35432, 0.}, {2.22376, 9.35432, 0.}}	{26.8565, 110.042, 100.}	inf	1073.	5380.64	{5290.06, 5465.9}	401.458	0.700201	newly	1153.	3238.04	{3170., 3316.}	180.836	0.448389	Reff	0.522297	0.407669	{0.406111, 0.409186}	21.9469	ar	71263.	84835.5	{84713., 84950.}	19.0456
15	R0	{1.99928, 7.1501, 0.1}	{2.13505, 8.31782, 0.}	{{2.13505, 8.31782, 0.}, {2.13505, 8.31782, 0.}}	{6.79086, 16.3315, 100.}	inf	2594.	3176.94	{3098.41, 3248.39}	22.4727	0.0879735	newly	1932.	2098.91	{2034., 2169.}	8.63923	0.0358651	Reff	0.446419	0.40509	{0.403817, 0.406374}	9.25797	ar	79602.	82992.2	{82812., 83124.}	4.25891
16	R0	{2.67398, 10.3134, 1.}	{2.64721, 11.9965, 0.}	{{2.64721, 11.9965, 0.}, {2.64721, 11.9965, 0.}}	{1.00103, 16.3197, 100.}	inf	4744.	7420.41	{7339.87, 7515.17}	56.4168	0.194265	newly	2584.	3555.91	{3493., 3618.}	37.6126	0.138616	Reff	0.275794	0.311849	{0.310489, 0.313279}	13.073	ar	91289.	91009.	{90911., 91104.}	0.30674
17	R0	{2.44634, 16.3226, 0.1}	{2.73517, 19.6025, 2.}	{{2.73517, 19.6025, 2.}, {2.73517, 19.6025, 2.}}	{11.8066, 20.0943, 1900.}	inf	11754.	14505.3	{14418.5, 14610.5}	23.407	0.0913345	newly	4470.	4637.84	{4560., 4714.}	3.76018	0.0159936	Reff	0.431632	0.389847	{0.387914, 0.39163}	9.68069	ar	88710.	91893.3	{91771., 92022.}	3.58846
18	R0	{2.02168, 10.1676, 0.1}	{2.02235, 10.6804, 0.}	{{2.02235, 10.6804, 0.}, {2.02235, 10.6804, 0.}}	{0.0333691, 5.04351, 100.}	inf	6854.	7376.02	{7278.95, 7462.83}	7.61621	0.0318584	newly	4020.	4130.89	{4053., 4213.}	2.79229	0.0119156	Reff	0.515609	0.525278	{0.524051, 0.526424}	1.87519	ar	80299.	80292.3	{80103., 80476.}	0.146179
19	R0	{2.89969, 15.009, 0.1}	{2.7499, 15.3124, 0.}	{{2.7499, 15.3124, 0.}, {2.7499, 15.3124, 0.}}	{5.16582, 2.02154, 100.}	inf	11247.	13088.1	{12978., 13202.}	16.3694	0.0658312	newly	4512.	5169.26	{5074., 5254.}	14.5669	0.0590249	Reff	0.292405	0.36202	{0.360313, 0.363737}	23.8079	ar	93349.	92037.7	{91920., 92170.}	1.40472
20	R0	{2.68933, 7.34928, 0.1}	{2.91001, 10.5991, 0.}	{{2.91001, 10.5991, 0.}, {2.91001, 10.5991, 0.}}	{8.20564, 44.2194, 100.}	inf	2277.	6046.11	{5961.32, 6120.39}	165.529	0.42409	newly	1542.	3133.68	{3065., 3197.}	103.222	0.307912	Reff	0.246316	0.240224	{0.238873, 0.24165}	2.47323	ar	91574.	93401.1	{93334., 93466.}	1.99525
21	R0	{2.15352, 9.28074, 0.1}	{2.38758, 11.1331, 1.}	{{2.38758, 11.1331, 1.}, {2.38758, 11.1331, 1.}}	{10.8685, 19.9593, 900.}	inf	5169.	6727.33	{6630.59, 6811.34}	30.1476	0.114414	newly	3172.	3486.15	{3414., 3558.}	9.90385	0.0409511	Reff	0.421315	0.374386	{0.372993, 0.375565}	11.1387	ar	83845.	87668.7	{87537., 87770.}	4.56039
22	R0	{2.21953, 9.04387, 0.1}	{2.68687, 12.2271, 1.}	{{2.68687, 12.2271, 1.}, {2.68687, 12.2271, 1.}}	{21.0558, 35.1979, 900.}	inf	4637.	7574.43	{7500.45, 7657.66}	63.3476	0.213097	newly	2827.	3566.01	{3506., 3619.}	26.1411	0.100815	Reff	0.397451	0.303824	{0.302146, 0.305283}	23.5568	ar	84856.	91413.5	{91316., 91510.}	7.72782
23	R0	{2.21987, 14.5347, 1.}	{2.76795, 19.6041, 1.}	{{2.76795, 19.6041, 1.}, {2.76795, 19.6041, 1.}}	{24.6897, 34.8782, 0.}	inf	9287.	13572.1	{13480.9, 13668.}	46.141	0.164766	newly	3912.	4325.98	{4256., 4395.}	10.5823	0.0436491	Reff	0.465951	0.364181	{0.362589, 0.365893}	21.8413	ar	85067.	92195.8	{92082., 92317.}	8.38019
24	R0	{2.84268, 17.9659, 1.}	{2.56757, 17.005, 0.}	{{2.56757, 17.005, 0.}, {2.56757, 17.005, 0.}}	{9.67771, 5.34863, 100.}	inf	14057.	12301.4	{12181.6, 12405.8}	12.4894	0.0579504	newly	4888.	4479.09	{4387., 4560.}	8.36559	0.0379827	Reff	0.351242	0.40502	{0.403231, 0.406721}	15.3109	ar	92836.	90125.5	{90002., 90270.}	2.91966
25	R0	{2.52706, 10.3679, 0.1}	{2.7499, 15.3124, 0.}	{{2.7499, 15.3124, 0.}, {2.7499, 15.3124, 0.}}	{8.81804, 47.6906, 100.}	inf	5201.	9664.74	{9576.02, 9762.26}	85.8247	0.269091	newly	2743.	3780.75	{3704., 3852.}	37.8327	0.139303	Reff	0.316085	0.313937	{0.312401, 0.315529}	0.685863	ar	89623.	92048.8	{91944., 92147.}	2.70662
26	R0	{2.62835, 19.1088, 1.}	{2.73517, 19.6025, 2.}	{{2.73517, 19.6025, 2.}, {2.73517, 19.6025, 2.}}	{4.06417, 2.58367, 100.}	inf	14411.	14508.6	{14408.7, 14598.1}	0.734216	0.00317343	newly	4701.	4634.6	{4560., 4700.}	1.51883	0.00667122	Reff	0.418354	0.389687	{0.38815, 0.391066}	6.85239	ar	90908.	91910.3	{91776., 92050.}	1.10252
27	R0	{2.76831, 19.4155, 2.}	{2.4452, 16.1758, 3.}	{{2.42687, 16.0321, 3.}, {2.51417, 16.7164, 3.}}	{11.6717, 16.6863, 50.}	inf	12467.	9886.75	{9788.93, 9966.42}	20.6967	0.100721	newly	3937.	3758.48	{3632., 3834.}	4.53442	0.020236	Reff	0.347063	0.404746	{0.383632, 0.411719}	16.6202	ar	92175.	88519.2	{88144., 89436.}	3.96618
28	R0	{2.96609, 16.8903, 1.}	{2.33968, 11.4691, 2.}	{{2.33968, 11.4691, 2.}, {2.33968, 11.4691, 2.}}	{21.119, 32.0966, 100.}	inf	11510.	6699.13	{6614.5, 6798.72}	41.7973	0.235085	newly	4186.	3399.74	{3323., 3479.}	18.7831	0.0904223	Reff	0.274037	0.389542	{0.388146, 0.391023}	42.1495	ar	94024.	86913.1	{86775., 87053.}	7.56282
29	R0	{2.17046, 14.39, 0.1}	{2.19372, 14.2723, 1.}	{{2.1255, 13.6009, 1.}, {2.315, 15.4658, 1.}}	{3.72311, 6.20076, 900.}	inf	10054.	10171.6	{9575.85, 11143.}	6.22354	0.0264845	newly	4398.	4409.55	{4324., 4483.}	1.12437	0.00487015	Reff	0.504914	0.509017	{0.474404, 0.529215}	5.0264	ar	84001.	84132.6	{82619., 86604.}	1.99542
30	R0	{2.88772, 12.4647, 1.}	{2.79531, 12.1249, 2.}	{{2.79531, 12.1249, 2.}, {2.79531, 12.1249, 2.}}	{3.20015, 2.72599, 100.}	inf	8175.	8087.02	{7994.79, 8171.08}	1.15475	0.00505359	newly	3775.	3813.48	{3721., 3899.}	1.61854	0.00696924	Reff	0.263591	0.284613	{0.283048, 0.286219}	7.97526	ar	93273.	92452.6	{92355., 92543.}	0.87959
31	R0	{2.46831, 8.14308, 0.1}	{2.17292, 6.16453, 0.}	{{2.17292, 6.16453, 0.}, {2.17292, 6.16453, 0.}}	{11.9675, 24.2973, 100.}	inf	3768.	1969.13	{1912.58, 2027.02}	47.7407	0.281943	newly	2494.	1599.68	{1545., 1649.}	35.8589	0.193001	Reff	0.317474	0.375989	{0.374962, 0.377102}	18.4314	ar	88762.	83825.1	{83717., 83921.}	5.56191
32	R0	{1.93236, 7.38987, 0.1}	{1.737, 5.43578, 0.}	{{1.737, 5.43578, 0.}, {1.737, 5.43578, 0.}}	{10.11, 26.4429, 100.}	inf	3204.	1752.03	{1685.45, 1810.37}	45.3173	0.26231	newly	2353.	1702.75	{1632., 1763.}	27.6349	0.140676	Reff	0.482588	0.542185	{0.540915, 0.543402}	12.3496	ar	77654.	70723.7	{70503., 70887.}	8.92461
33	R0	{2.82251, 17.6954, 1.}	{2.51743, 15.191, 3.}	{{2.51743, 15.191, 3.}, {2.51743, 15.191, 3.}}	{10.8089, 14.1528, 200.}	inf	15003.	12321.8	{12206.3, 12410.5}	17.8709	0.0855108	newly	5209.	4958.72	{4869., 5034.}	4.80476	0.0214234	Reff	0.365938	0.42223	{0.420488, 0.423818}	15.3827	ar	92745.	89504.9	{89371., 89632.}	3.49351
34	R0	{1.87083, 12.3994, 1.}	{2.07206, 15.0886, 4.}	{{2.07206, 15.0886, 4.}, {2.07206, 15.0886, 4.}}	{10.7562, 21.6882, 300.}	inf	8229.	10669.3	{10557.3, 10773.6}	29.655	0.112776	newly	4286.	4482.42	{4408., 4559.}	4.58283	0.0194152	Reff	0.630488	0.582813	{0.580949, 0.584635}	7.56172	ar	76282.	81531.5	{81341., 81764.}	6.88175
35	R0	{2.95779, 16.3003, 1.}	{2.5684, 13.7324, 1.}	{{2.5684, 13.7324, 1.}, {2.5684, 13.7324, 1.}}	{13.1649, 15.7535, 0.}	inf	14081.	10742.3	{10620.6, 10841.9}	23.7106	0.117547	newly	5272.	4664.9	{4594., 4730.}	11.5156	0.0531621	Reff	0.314472	0.379016	{0.377411, 0.380586}	20.5245	ar	93873.	90116.1	{89972., 90253.}	4.00212
36	R0	{2.18718, 14.261, 3.}	{2.29535, 15.2438, 4.}	{{2.2143, 14.4309, 4.}, {2.41197, 16.4137, 4.}}	{4.94546, 6.8918, 33.3333}	inf	9571.	10819.	{10033.2, 11876.7}	13.0395	0.0519007	newly	4189.	4404.06	{4290., 4527.}	5.13392	0.0216459	Reff	0.488835	0.478921	{0.4504, 0.49901}	4.25465	ar	84379.	86032.2	{84494., 88127.}	1.96186
37	R0	{2.44912, 9.31118, 0.1}	{2.22794, 8.48499, 1.}	{{2.19024, 8.25876, 1.}, {2.19024, 8.25876, 1.}}	{10.8414, 12.3761, 900.}	inf	4364.	3982.71	{3694.87, 3862.85}	16.6712	0.0725472	newly	2540.	2553.09	{2428., 2575.}	4.04213	0.0163724	Reff	0.332493	0.387954	{0.393552, 0.397045}	18.928	ar	88442.	84662.4	{84020., 84288.}	4.84086
38	R0	{2.05949, 5.72514, 0.1}	{2.70795, 10.355, 0.}	{{2.70795, 10.355, 0.}, {2.70795, 10.355, 0.}}	{31.4867, 80.8682, 100.}	inf	1447.	5342.61	{5263.51, 5418.95}	269.22	0.567257	newly	1229.	2849.62	{2789., 2906.}	131.865	0.365175	Reff	0.397749	0.27447	{0.273142, 0.275684}	30.9943	ar	81587.	91634.8	{91547., 91728.}	12.3154
39	R0	{1.75505, 8.52404, 0.1}	{1.87584, 10.2502, 2.}	{{1.87584, 10.2502, 2.}, {1.87584, 10.2502, 2.}}	{6.88266, 20.2508, 1900.}	inf	4589.	6110.54	{6016.74, 6202.81}	33.1563	0.124334	newly	3152.	3597.92	{3502., 3679.}	14.1472	0.0573992	Reff	0.605615	0.569947	{0.568224, 0.57151}	5.88959	ar	70925.	75929.3	{75673., 76138.}	7.05579
40	R0	{2.24724, 13.7861, 1.}	{2.04871, 11.6322, 3.}	{{2.04871, 11.6322, 3.}, {2.04871, 11.6322, 3.}}	{8.8346, 15.6236, 200.}	inf	8230.	6308.93	{6193.72, 6394.57}	23.3422	0.115472	newly	3630.	3259.84	{3183., 3333.}	10.1972	0.0467792	Reff	0.451088	0.490848	{0.489559, 0.492123}	8.81424	ar	85257.	80977.1	{80791., 81138.}	5.01996
41	R0	{1.77635, 8.62406, 0.1}	{1.96373, 10.9366, 4.}	{{1.96373, 10.9366, 4.}, {1.96373, 10.9366, 4.}}	{10.5483, 26.8153, 3900.}	inf	4190.	6126.96	{6034.66, 6221.69}	46.2281	0.165002	newly	2830.	3369.9	{3291., 3461.}	19.0777	0.075756	Reff	0.557952	0.525775	{0.524235, 0.527137}	5.76682	ar	72941.	78661.4	{78450., 78848.}	7.84246
42	R0	{1.80397, 5.4932, 1.}	{1.81768, 5.68021, 1.}	{{1.81768, 5.68021, 1.}, {1.81768, 5.68021, 1.}}	{0.760022, 3.40431, 0.}	inf	1509.	1331.86	{1283.86, 1385.42}	11.739	0.0544214	newly	1451.	1221.93	{1169., 1281.}	15.787	0.0749065	Reff	0.511444	0.497362	{0.496235, 0.498265}	2.75333	ar	73232.	73888.	{73737., 74004.}	0.895838
43	R0	{2.83096, 16.7077, 0.1}	{2.93978, 19.9817, 0.}	{{2.93978, 19.9817, 0.}, {2.93978, 19.9817, 0.}}	{3.84376, 19.5959, 100.}	inf	13176.	16927.8	{16814., 17027.7}	28.4742	0.108811	newly	4750.	5304.6	{5213., 5379.}	11.6758	0.0479268	Reff	0.342093	0.370313	{0.368367, 0.372196}	8.24911	ar	92740.	93618.2	{93524., 93724.}	0.946938
44	R0	{2.73678, 11.6021, 1.}	{2.99939, 13.7076, 1.}	{{2.99939, 13.7076, 1.}, {2.99939, 13.7076, 1.}}	{9.5957, 18.1478, 0.}	inf	5400.	7537.52	{7461.5, 7620.99}	39.5837	0.14482	newly	2650.	3186.96	{3117., 3253.}	20.2626	0.0800777	Reff	0.263771	0.237536	{0.236252, 0.238906}	9.94615	ar	92003.	94039.3	{93970., 94127.}	2.21332
45	R0	{2.14235, 6.98601, 0.1}	{2.13505, 8.31782, 0.}	{{2.13505, 8.31782, 0.}, {2.13505, 8.31782, 0.}}	{0.340824, 19.0639, 100.}	inf	1905.	3174.49	{3103.1, 3263.69}	66.64	0.221711	newly	1420.	2099.03	{2041., 2166.}	47.819	0.169617	Reff	0.389458	0.405063	{0.403684, 0.406298}	4.00687	ar	82974.	83000.	{82857., 83130.}	0.104599
46	R0	{2.23409, 9.21289, 0.1}	{2.53075, 11.6597, 0.}	{{2.53075, 11.6597, 0.}, {2.53075, 11.6597, 0.}}	{13.2788, 26.5587, 100.}	inf	5803.	8581.78	{8489.49, 8694.48}	47.8852	0.169906	newly	3539.	4269.5	{4187., 4356.}	20.6414	0.0814402	Reff	0.407766	0.358272	{0.356278, 0.359962}	12.138	ar	85163.	89630.3	{89510., 89752.}	5.24559
47	R0	{2.12258, 8.15265, 0.1}	{2.19024, 8.25876, 1.}	{{2.19024, 8.25876, 1.}, {2.19024, 8.25876, 1.}}	{3.18751, 1.30154, 900.}	inf	3887.	3782.34	{3707.62, 3859.61}	2.78031	0.0122909	newly	2681.	2502.74	{2432., 2562.}	6.64901	0.0299769	Reff	0.420441	0.395133	{0.393755, 0.396408}	6.01935	ar	82690.	84173.1	{84050., 84289.}	1.79357
48	R0	{2.71771, 10.4636, 0.1}	{2.73581, 10.6512, 0.}	{{2.74056, 10.6768, 0.}, {2.74056, 10.6768, 0.}}	{0.998584, 2.24018, 100.}	inf	6665.	6045.72	{5981.42, 6141.52}	9.29153	0.0426899	newly	3585.	3149.06	{3077., 3226.}	12.1601	0.0564258	Reff	0.28718	0.276088	{0.273732, 0.276553}	4.46367	ar	91716.	91886.9	{91848., 92045.}	0.318014
49	R0	{2.44369, 6.66477, 0.1}	{2.77394, 8.24535, 0.}	{{2.77394, 8.24535, 0.}, {2.77394, 8.24535, 0.}}	{13.5144, 23.7155, 100.}	inf	1262.	2233.12	{2175.58, 2289.3}	76.951	0.247773	newly	921.	1390.37	{1337., 1442.}	50.9631	0.1787	Reff	0.285399	0.232425	{0.231267, 0.233287}	18.5613	ar	88827.	92261.4	{92197., 92316.}	3.86636
50	R0	{2.77383, 17.9775, 0.1}	{2.6893, 17.4298, 0.81}	{{2.64334, 17.2462, 0.}, {2.88523, 18.2127, 1.}}	{4.57356, 3.54359, 748.}	inf	11601.	11128.1	{10967.2, 11407.8}	4.07656	0.0181167	newly	3992.	3922.63	{3830., 4006.}	1.9246	0.00848788	Reff	0.332083	0.3505	{0.303599, 0.362904}	8.8108	ar	92334.	91379.2	{90848., 93186.}	1.38669
